# Impact of Reverberation on Speech Perception in Noise in Bimodal/Bilateral Cochlear Implant Users with and without Residual Hearing

**DOI:** 10.3390/jcm13175269

**Published:** 2024-09-05

**Authors:** Clara König, Uwe Baumann, Timo Stöver, Tobias Weissgerber

**Affiliations:** 1 Audiological Acoustics, ENT Department, University Hospital, Goethe University Frankfurt, 60590 Frankfurt am Main, Germany; uwe.baumann@unimedizin-ffm.de (U.B.); weissgerber@med.uni-frankfurt.de (T.W.); 2 ENT Department, University Hospital, Goethe University Frankfurt, 60590 Frankfurt am Main, Germany; stoever@med.uni-frankfurt.de

**Keywords:** cochlear implant, electric–acoustic stimulation, reverberation, speech perception, spatial release from masking

## Abstract

(1) **Background**: The aim of the present study was to assess the impact of reverberation on speech perception in noise and spatial release from masking (SRM) in bimodal or bilateral cochlear implant (CI) users and CI subjects with low-frequency residual hearing using combined electric–acoustic stimulation (EAS). (2) **Methods**: In total, 10 bimodal, 14 bilateral CI users and 14 EAS users, and 17 normal hearing (NH) controls, took part in the study. Speech reception thresholds (SRTs) in unmodulated noise were assessed in co-located masker condition (S0N0) with a spatial separation of speech and noise (S0N60) in both free-field and loudspeaker-based room simulation for two different reverberation times. (3) **Results**: There was a significant detrimental effect of reverberation on SRTs and SRM in all subject groups. A significant difference between the NH group and all the CI/EAS groups was found. There was no significant difference in SRTs between any CI and EAS group. Only NH subjects achieved spatial release from masking in reverberation, whereas no beneficial effect of spatial separation of speech and noise was found in any CI/EAS group. (4) **Conclusions**: The subject group with electric–acoustic stimulation did not yield a superior outcome in terms of speech perception in noise under reverberation when the noise was presented towards the better hearing ear.

## 1. Introduction

Cochlear implants (CIs) can help many users with severe to profound sensorineural hearing loss to achieve good speech perception in quiet within a range that is often comparable to that of individuals with normal hearing (NH) [1]. However, CI users oftentimes struggle to understand speech in everyday listening conditions that comprise noise sources and/or reverberation [2,3].

For NH listeners, speech perception in noisy environments improves when the signal and noise sources are spatially separated compared to co-located speech and masker conditions. This effect is called spatial release from masking (SRM) which is primarily caused by binaural (i.e., spatial) hearing (interaural level differences and interaural time differences) in combination with monaural better ear effects [4,5]. Previous studies have shown that the improvement in speech perception using SRM is reduced or even absent in bilateral CI users or bimodal CI users using a hearing aid in the contralateral ear [6,7].

Another effect which deteriorates speech perception in NH as well as in CI users is reverberation. While speech perception in quiet is hardly affected by reverberation in people with normal hearing, there is a significant reduction in speech perception in cochlear implant users [2,8,9,10]. On the other hand, it was reported that reverberation has comparable detrimental effects on speech perception in noise for both NH and CI subjects [11,12]. However, it has to be noted that CI users are by far more affected by reverberation in everyday life since their performance (measured as speech reception threshold, SRT, measured in dB SNR) in the free-field is already up to 10 dB worse than in NH [11]. Studies investigating the effect of reverberation on speech perception in CI users were oftentimes conducted in simulated reverberation via headphone presentation or by using the direct audio input of the CI [3,8,10,13,14,15]. Furthermore, oftentimes CI listening with vocoder simulation instead of testing real CI subjects was performed [2,7,8,10,13,14,15,16,17].

Only a few studies investigated the effect of reverberation on speech perception in CI recipients using a real sound field by means of a loudspeaker array, allowing every tested person to use their individual head-related transfer functions [11,12,18]. It was shown that the impact of reverberation on SRTs in CI users was weaker than in studies using less complex room simulation methods without real loudspeakers. Therefore, the aim of the present study was to compare the impact of reverberation on speech perception in noise and SRM for different groups of CI users with and without residual hearing in one or both ears in a loudspeaker-based sound reproduction setup. 

A special focus will be placed here on the group with combined electric–acoustic stimulation (EAS), which has been insufficiently studied in the past since many of the aforementioned studies only compared speech perception performance between NH listeners, bimodal CI listeners and bilateral CI listeners. EAS is a well-accepted therapeutic treatment for CI users with residual hearing in the low frequencies but severe to profound hearing loss in the high frequencies [19]. The unilateral combination of the electric stimulation of the high frequencies via a CI and acoustic stimulation of the low frequencies via a hearing aid enables users to achieve better speech perception in quiet [20,21], in noise [20,21,22,23,24,25,26], and better sound localisation [26,27] than electric-only stimulation does. It is assumed that the benefit of the EAS listeners comes from access to frequency fine structure and F0 information, which facilitates the identification of low-frequency acoustic landmarks such as the onset of voicing and the syllable structure [28,29]. In a study with simulated CI and EAS listening, the largest SRM that was closest to the NH listeners was found in the group of bilateral EAS users [7]. To the knowledge of the authors, no studies comparing speech perception in reverberation between CI and EAS in loudspeaker-based sound reproduction setups have been published so far. 

## 2. Materials and Methods

### 2.1. Subject Demographics

A total of 10 unilateral CI users with a hearing aid in the contralateral ear (i.e., bimodal CI users; 6 female, mean age: 52.5 ± 19.2 years), 14 bilateral CI users (8 female, mean age: 49.9 ± 16.4 years) and two groups of EAS users (bimodal group with hearing aid or CI in contralateral ear: n = 8, 5 female, mean age: 60.1 ± 8.5 years; bilateral group: n = 6, 3 female, mean age: 61.5 ± 12.4 years) took part in the study. All participants were implanted with MED-EL (Innsbruck, Austria) devices with either SONNET or OPUS 2 (or DUET 2 for EAS) sound processors. The minimum experience with their CI was 2.4 months and the mean duration of cochlear implant use was 5.2 years, ranging from 2.4 months to 17.4 years. The inclusion criterion was that the individual monosyllable score in quiet (Freiburg monosyllables at 65 dB sound pressure level, [30]) and in the ipsilateral ear (or in cases with bilateral CI/EAS in the better hearing ear) had to be better than 50%. For lower speech perception scores in quiet, no measurement of speech reception thresholds in noise would be feasible. All subjects were tested with their everyday fitting map. The proper fitting of sound processors and hearing aids was assessed using aided free-field audiometry (hearing thresholds) and speech audiometry (Freiburg monosyllable score). In case of the use of the SONNET sound processor, the microphone directionality was set to omnidirectional. Detailed information on all CI/EAS users can be found in Table 1, Table 2, Table 3 and Table 4. 

A group of 17 subjects (14 female, mean age: 26.7 ± 8.0 years) with normal hearing (i.e., no pure-tone hearing loss >25 dB HL in any test frequency between 0.125 and 8 kHz) served as a control group.

### 2.2. Speech Perception Test in Reverberation

Testing was performed in an anechoic chamber using 128 custom-built loudspeakers which were mounted in the horizontal plane at a height of 1.20 m. The distance between adjacent loudspeakers was 8.6 cm. Further information about the loudspeakers setup is given in [31]. Speech tests were conducted in free-field conditions and under loudspeaker-based reverberation simulation. Reverberation was created with a custom room simulation tool combining the nearest speaker method for early reflections and a feedback delay network to simulate late reflections [32]. For the room simulation in the present study, a three-dimensional model of a lecture hall simulated with two different degrees of absorption corresponding to reverberation times of 0.35 s and 0.51 s was used [32]. 

Speech perception in noise was measured with the German Matrix Test (Oldenburg Sentence Test, OlSa, [33]). The noise level was kept constant at 65 dB SPL. The speech level was adaptively changed to measure the speech reception threshold (SRT) for a 50% correct word understanding. The speech signal was always presented from frontal direction (0° azimuth). The noise signal was the continuous noise of the OlSa test which was either presented from the front (i.e., co-located masker at 0°, S0N0) or from the side (either from +60° or −60° azimuth, S0N60). In the S0N60 condition, the noise was always presented on the side of the CI/EAS system in the case of bimodal CI/EAS users or on the side of the better ear in the case of bilateral CI/EAS fitting. Both spatial configurations of S0N0 and S0N60 were tested in free-field and in reverberation with 0.35 s and 0.51 s reverberation time, respectively. One OlSa list with 20 sentences each was used for each test condition. Prior to testing, one practice list was presented to the subject to familiarise the subject with the test procedure and the speech material. The test was conducted in a closed-set mode and the order of the test lists was randomised. 

All study tests in this prospective study were performed for each subject on a single appointment. The test duration was approximately 90–120 min.

### 2.3. Statistics

Boxplots and median values were used for descriptive analyses throughout the manuscript. Nonparametric tests were utilised for statistical analyses of SRT differences (group differences: Kruskal–Wallis H test; impact of test condition within subject groups: Friedman test). Post hoc tests were performed using the Mann–Whitney U-test (group differences) or the Wilcoxon test (within-group). Correlations were tested via Spearman rank correlation. A *p* value < 0.05 was considered significant. IBM SPSS Statistics 27 (IBM, Armonik, New York, NY, USA) was used for the analysis.

## 3. Results

### 3.1. Impact of Reverberation on SRTs

**Figure 1:**
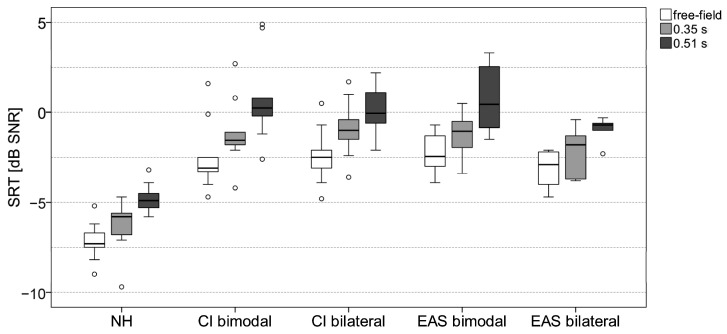
Boxplots of the SRT measurement results for all five subject groups in the S0N0 condition in free-field (white boxes) and reverberation with reverberation time of 0.35 s (light grey boxes) and 0.51 s (dark grey boxes). Each outlier that is more than 1.5 times the interquartile range is indicated with a circle.

The boxplots of the SRT measurement results in the S0N0 condition in free-field and reverberation are shown for all participants in Figure 1. There was a significant effect of reverberation in all subject groups (NH: Χ^2^ = 28.4, *p* < 0.001; CI bimodal: Χ^2^ = 20.0, *p* < 0.001; CI bilateral: Χ^2^ = 23.3, *p* < 0.001; EAS bimodal: Χ^2^ =8.9, *p* = 0.012; EAS bilateral: Χ^2^ = 10.3, *p* = 0.006; all df = 2; Friedman test) showing higher SRTs with increasing reverberation time. The median SRT difference between the free-field condition and test condition with the highest reverberation time was between 2.2 dB (bilateral EAS group) and 3.4 dB (bimodal CI group). 

There was also a significant effect of subject group on SRTs (free-field: H = 35.5; 0.35 s reverberation: H = 36.3; 0.51 s reverberation: H = 37.2; all *p* < 0.001; all df = 4; Kruskal–Wallis H test). Non-parametric post hoc tests found significant differences in SRT between the NH group and all CI/EAS groups (all *p* < 0.001). The SRTs in the NH group were 4.2–5.2 dB better than the SRTs in the CI and EAS groups. However, no significant difference in the SRTs was found between any CI/EAS group in test condition S0N0. 

**Figure 2:**
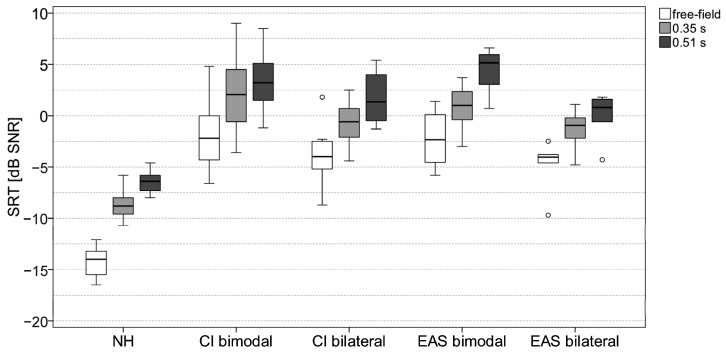
Boxplots of the SRT measurement results for all five subject groups in the S0N60 condition in free-field (white boxes) and reverberation with reverberation time of 0.35 s (light grey boxes) and 0.51 s (dark grey boxes). Each outlier that is more than 1.5 times the interquartile range is indicated with a circle.

The boxplots of the SRT measurements in the S0N60 condition of all participants in free-field and reverberation are shown in Figure 2. There was a significant effect of reverberation in all subject groups (NH: Χ^2^ = 30.5, *p* < 0.001; CI bimodal: Χ^2^ = 18.2, *p* < 0.001; CI bilateral: Χ^2^ = 26.1, *p* < 0.001; EAS bimodal: Χ^2^ = 14.0, *p* < 0.001; EAS bilateral: Χ^2^ = 12.0, *p* = 0.002; all df = 2). The SRTs increased with increasing reverberation time. The median SRT difference between the free-field condition and test condition with the longest reverberation time was between 4.9 dB (bilateral EAS group) and 7.6 dB (bimodal CI group). 

There was also a significant effect of subject group on SRTs (free-field: H = 36.5, *p* < 0.001; 0.35 s reverberation: H = 37.7, *p* < 0.001; 0.51 s reverberation: H = 40.1, *p* < 0.001). Post hoc tests found significant differences in SRT between the NH group and all the CI/EAS groups (all *p* < 0.001). The SRTs in the NH group were 7.2–11.8 dB better than the SRTs in the CI and EAS groups. This was the same as the condition S0N0; no significant difference in SRTs was found between any CI and EAS group in test condition S0N60.

### 3.2. Impact of Reverberation on Spatial Release from Masking

**Figure 3:**
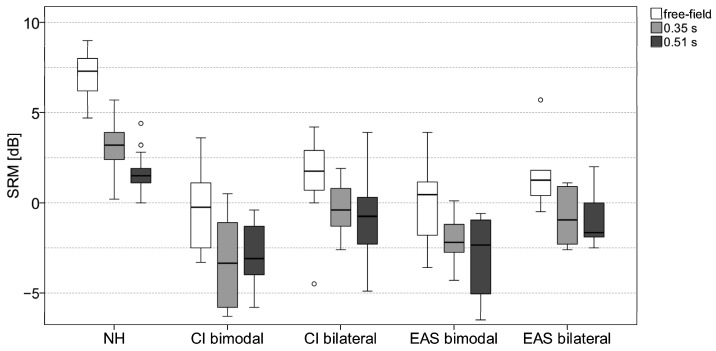
Boxplots of spatial release from masking (SRM, individual SRT difference between S0N0 and S0N60 results) for all five subject groups in free-field (white boxes) and reverberation with reverberation time of 0.35 s (light grey boxes) and 0.51 s (dark grey boxes). Each outlier that is more than 1.5 times the interquartile range is indicated with a circle.

The boxplots of spatial release from masking (SRM, i.e., individual SRT difference between S0N0 and S0N60) are shown in Figure 3. There was a significant effect of reverberation on SRM in the NH group (Χ^2^ = 30.2, *p* < 0.001), the bimodal groups with CI (Χ^2^ = 13.3, *p* = 0.001) and EAS (Χ^2^ = 14.3, *p* = 0.001), and in the bilateral groups with CI (Χ^2^ = 9.0, *p* = 0.011) and EAS (Χ^2^ = 12.3, *p* = 0.002).

There was also a significant effect of subject group on the SRM (free-field: H = 37.6, *p* < 0.001; 0.35 s reverberation: H = 38.9, *p* < 0.001; 0.51 s reverberation: H = 30.3, *p* < 0.001). Post hoc tests found significant differences in SRM between the NH group and all CI/EAS groups (all *p* < 0.001). SRM in the NH group was 2.3–7.6 dB higher than SRM in the CI and EAS groups.

No significant difference in SRM was found between any CI and EAS group. It should be noted, that only in the bilateral CI and EAS groups, the majority of subjects could benefit from a spatial separation of speech in noise to improve speech perception (but only under free-field conditions). In contrast, approximately 50% of subjects in the bimodal groups had no SRM at all (i.e., a SRM score lower or equal 0 dB).

### 3.3. Impact of Acoustic Hearing on SRT and SRM

For the bimodal CI and EAS groups, correlations were calculated between the pure-tone average (PTA, frequencies 0.5/1/2/4 kHz) or the low-frequency pure-tone average (PTA_low_, frequencies 0.125/0.25/0.5 kHz) and SRT/SRM scores. There was no significant correlation between PTA or PTA_low_ and SRT or SRM scores in any subject group.

## 4. Discussion

### 4.1. Impact of Reverberation on SRTs

All subject groups showed a deterioration in SRTs in continuous noise with increasing reverberation time in both co-located S0N0 and spatially separated S0N60 conditions assessed in a loudspeaker-based room simulation setup. The detrimental effect of reverberation on SRT was 2.2–3.4 dB in the S0N0 condition and 4.9–7.6 dB in the S0N60 condition, depending on subject group. The detrimental effect of reverberation on SRTs was comparable in all subject groups. However, the baseline (i.e., SRT in free-field) was considerably higher (worse) in all CI groups, especially in the S0N60 condition (up to 12 dB).

To the knowledge of the authors, this is the first study that assessed the impact of reverberation on SRTs in noise in a population of EAS in comparison with the NH and CI groups using a loudspeaker-based sound reproduction setup. Helms-Tillery et al. investigated the effect of reverberation on speech perception in EAS using vocoder studies in NH subjects [17]. They found a significant “EAS effect” (better speech perception) in reverberation compared to simulated CI.

Kokkinakis and Loizou [8] reported for CI users a decrease in word recognition from 84% in an anechoic condition to 20% in reverberation (reverberation time: 1.0 s) at a source–receiver distance of 1 m. Kokkinakis et al. [34] extended this study by various reverberation times. They found that mean word recognition performance decreased exponentially with increasing reverberation time. In the present study, the relationship between reverberation time and SRT was rather linear. It must be taken into account that the method of room simulation differs significantly in many of the studies. Kokkinakis et al. used the static filtering of non-individualised head-related transfer functions (i.e., binaural and head-phone based approach). Differences in sound reproduction could have a higher impact on the results. Many studies did not take place in a real room or loudspeaker-based room simulation, but were presented via a direct audio output (e.g., [8,10,13,14,15,34]), or, the participants did not wear their own processors but research processors, which may have varied from their everyday processor (e.g., [8,13,15,34]). It is assumed that by using the user’s everyday CI system in a real sound field, more realistic spatial cues and natural reverberation can be included to reach results closer to real-life situations.

It should also be considered that reverberation is not the only room-acoustic parameter. In the present study, the reverberation time was modulated by changing the absorption properties of the surfaces inside the room while keeping the room geometry and the source and listener positions constant. Therefore, an increase in reverberation time goes along with a decrease in the direct-to-reverberant energy ratio (DRR). This means that the level of reverberation in relation to the direct sound is also increased. Thus, there were two acoustic parameters which could account for the deterioration in SRT.

In a real room, the reverberation time is almost place-independent, whereas the DRR is defined by the distance between source and listener. The influence of the distance between the talker and CI users was investigated by Kressner et al. [12] in a loudspeaker-based reproduction setup with three different room sizes and different receiver positions (1 m, 3 m, 6 m). They found a significant effect of the source–receiver distance, but no effect of the reverberation time on speech perception. Furthermore, no effect of the number of late reflections on speech perception was found. For a small source–receiver distance of 1 m (i.e., high DRRs), all participants except one showed speech perception scores better than 90%, even in a simulation of a large room (reverberation time: 1.7 s). These results contradict the data of Kokkinakis and Loizou [8] and Kokkinakis et al. [34], with a high impact of reverberation also at a small source–receiver distance, but measured with a binaural sound reproduction method. In the model of Kressner et al., an effect became significant with a source–receiver distance of 3 m and particularly worse in the big room auditorium simulation with a source-receiver distance of 6 m.

Badajoz-Davila et al. [18] also reported that speech perception does not necessarily decay with increasing reverberation time. The data were also obtained using loudspeaker-based sound reproduction. Speech perception in a smaller but more reflective room (reverberation time: 1.55 s) was more affected than in a bigger room (car park) with a higher reverberation time of 2.42 s. Badajoz-Davila et al. [18] assume that the quite strong effect of reverberation on speech perception in CI users that was observed in previous studies [2,3,8,14,34] is caused since small reverberant rooms were considered, containing an unrealistically high amount of reverberation.

In the present study, results close to everyday performance were expected since loudspeaker-based room simulation was used, which was shown to be beneficial compared to binaural sound reproduction methods in CI users. However, further studies should also assess the effect of DRR and reverberation time independently.

### 4.2. Impact of Reverberation on Spatial Release from Masking

Spatial release from masking as the effect of spatial separation of speech and continuous noise (measured as SRT difference between S0N0 and S0N60) was investigated for free-field conditions and two different reverberation times. In contrast to other studies, the separation was only 60° (i.e., no maximum head shadow effect as for 90°) and the noise was presented to the better hearing ear. A significant effect of reverberation on SRM was found for all subject groups. However, it has to be noted that even in free-field conditions, considerable differences in SRM between NH and all CI groups were found. The mean SRM in the NH group was higher than 7 dB, whereas in the bilateral CI and EAS groups only 75% of the subjects had a small SRM at all (median: 1.25–1.75 dB). In the bimodal groups, only 50% of the subjects achieved a small SRM in free-field conditions. No beneficial effect of EAS use on SRM was found.

The results are in line with data from Gifford et al. (2014). They found no beneficial effect of hearing preservation on SRM [6]. As in our study, the highest amount of SRM was in the bilateral CI group. Williges et al. [7] used vocoder studies to assess SRM in simulated CI and EAS subjects. SRM in the bilateral CI group was about 6 dB lower than in NH, which is in line with the data presented in our study. In contrast to the present results and the data reported in Gifford et al., a beneficial effect of EAS on SRM compared to bilateral CI was found.

SRM was severely deteriorated by reverberation in the NH group, showing decreasing SRM with increasing reverberation time. Rennies and Kidd [35] found that increasing reverberation not only leads to decreased SRM, but also to a strong deterioration of spatial release from listening effort.

None of the EAS or CI groups achieved any SRM in reverberation at all. The reason is probably that the noise was presented to the better ear. Since the subjects could benefit from the head shadow effect only in the poorer ear, SRM was already small in the free-field. By increasing diffusiveness due to reverberation, the head shadow effect was further diminished. It could be hypothesised that SRM in CI or EAS users would be higher in a symmetric noise/reverberation setup, where the better ear effect would be useable. The study of Weissgerber et al. [36] investigated SRM in a symmetric diffuse noise condition, showing that SRM in bimodal and the bilateral CI groups was comparable (2.1–2.2 dB) and close to NH (2.9 dB).

It has to be noted that the noise used in the present study was continuous noise. Further studies using temporally modulated noise were of interest to assess the combined effect of glimpsing and SRM in EAS vs. electric CI stimulation only.

### 4.3. Impact of Subject Group

There was a significant effect of subject group in all test conditions. However, post hoc tests revealed that only the results from the NH group were significantly different (i.e., better) compared to all EAS/CI groups, whereas no significant differences between the EAS or CI subject groups were found in any test condition.

These findings are in contradiction to other previous studies. The average EAS benefit in free-field is reported to be in the range of 5% to 30% in quiet [37] and 26% in noise [38]. Rader et al. [25] compared bilateral CI users and bimodal EAS users in the co-located masker condition S0N0 and in a diffuse multi-source noise field condition. In all test conditions, the EAS subject group demonstrated a significantly better outcome than the bilateral CI group. Some top-performing EAS users came very close to the results of normal-hearing listeners.

Turner et al. [39] could not find better SRTs in simulated EAS listening compared to simulated CI stimulation in a continuous noise condition, whereas in a more complex scenario with competitive talkers, better SRTs were found in the EAS group. The results were confirmed in a small sample of EAS users compared to a group of CI users.

In the present study, the noise in the spatially separated noise condition S0N60 was directed towards the better ear. In the conditions in either noise or reverberation, there was a clinically relevant tendency of better SRTs in the bilateral groups (either EAS or CI) compared to the bimodal CI/EAS groups. For all EAS/CI subject groups, pairwise comparisons revealed a significantly lower monosyllable recognition score in the worse ear. However, this difference was larger in the bimodal CI (28.5%) and EAS (36.4%) groups than in the bilateral CI (12.2%) and EAS (15.8%) groups.

The mean age of the groups with bimodal or bilateral CI stimulation was almost 10 years lower than in the respective EAS groups. It is known that speech perception scores in noise decrease with increasing age, even in normal hearing subjects and when partialling out a potential age-related hearing loss [40]. Furthermore, a recent study reported that age is the only predictive factor for speech perception performance in noise in CI subjects [41]. Therefore, the EAS effect found in previous studies was probably absent in the present study because the age of the CI subject groups was lower. However, testing age-matched subject groups is oftentimes hard to realise in studies with EAS subjects since this is a rather rare subject population.

It has to be noted that in the spatially separated speech in the noise condition, the best performing subject showing SRTs and SRM closest to the NH subjects was in the bilateral EAS group with exceptionally good residual hearing in both ears (subject EAS_BL6 in Table 4).

### 4.4. Limitation of the Study

The presented work is not without limitations. As the group of EAS users is generally a small population, the number of cases tested in this study is correspondingly low. Studies with larger case numbers would be desirable, so that a stronger correlation between residual hearing and hearing performance in the reverberation could possibly be shown. This was also the reason why it was not possible to match the age of the different study groups (see previous section). Furthermore, the test in the spatially separated condition was always conducted presenting the noise on the EAS ear. Further experiments with the presentation of noise in the contralateral ear were of interest, as SRM would probably be higher in such a test condition.

## 5. Conclusions

The test procedure, which employed loudspeaker-based room simulation, revealed that reverberation has a detrimental impact on SRT across all subject groups. Only NH subjects achieved spatial release from masking in reverberation, whereas no beneficial effect of spatial separation of speech and noise was found in any CI/EAS group, when noise was presented towards the better hearing ear.

No beneficial effect of combined electric–acoustic stimulation compared to electric stimulation on speech perception in reverberation was found. Further studies on this research question using age-matched subject groups are desirable.

## Figures and Tables

**Table 1:** Data of the bimodal subject group. FMS: Freiburg monosyllable score. PTA: pure-tone average of frequencies 0.5/1/2/4 kHz. Used hearing aids were from manufacturers Unitron (Kitchener, ON, Canada), Phonak (Stäfa, Switzerland), Audio Service (Löhne, Germany), ReSound (Ballerup, Denmark), and Widex (Lynge, Denmark).

ID	Implant Type (Ear)	Age [yrs]	Sound Processor	CI Listening Experience [yrs]	Hearing Aid Type	FMS CI [%]	FMS HA [%]	PTA (Contralateral) [dB HL]
BM 1	Concerto Flex24 (right)	57.3	OPUS 2	4.2	Unitron Moda 2	95	95	35.0
BM 2	Concerto Flex24 (right)	73.6	OPUS 2	3.6	Phonak Dalia SC	75	25	88.8
BM 3	Pulsar Standard (left)	23.6	OPUS 2	16.3	Phonak Naida S	90	0	106.3
BM 4	Concerto Flex28 (right)	71.9	OPUS 2	4.3	Phonak Naida S3	55	85	47.5
BM 5	Synchrony Flex28 (left)	67.6	SONNET	2.2	Audio Service Mezzo Duo	70	0	77.5
BM 6	Synchrony Flex28 (left)	34.7	SONNET	2.0	ReSound Preza	80	40	103.8
BM 7	Concerto Flex28 (left)	73.0	OPUS 2	5.0	Phonak Naida S V UP	85	55	77.5
BM 8	Synchrony Flex28 (left)	33.0	OPUS 2	2.1	Phonak Naida 5 S1	65	60	61.3
BM 9	Sonata Flex24 (right)	55.2	OPUS 2	7.1	Widex Inteo	60	30	101.3
BM 10	Synchrony Flex28 (left)	35.7	SONNET	1.6	Phonak Naida Q50-SP	80	80	43.8

**Table 2:** Data of the bilateral subject group. FMS: Freiburg monosyllable score, ipsilateral ear for noise presentation in S0N60 test condition is indicated bold.

ID	Implant Type (Left/Right)	Age [yrs]	Sound Processor	CI Listening Experience Left [yrs]	CI Listening Experience Right [yrs]	FMS Left [%]	FMS Right [%]
BL 1	Concerto Flex28/FlexSoft	63.3	OPUS 2	4.1	5.2	70	**85**
BL 2	Concerto FLEXsoft/FLEX28	57.6	OPUS 2	5.0	4.0	**95**	95
BL 3	Concerto FLEXsoft/FLEX28	70.1	OPUS 2	4.2	5.4	**85**	85
BL 4	Sonata FLEXsoft Pulsar Standard	50.1	OPUS 2	6.3	9.9	**90**	80
BL 5	Concerto FLEX24	59.5	OPUS 2	3.7	2.9	75	**80**
BL 6	Concerto FLEX28 C40+ Standard	38.1	OPUS 2	4.4	13.6	85	**90**
BL 7	Sonata Standard Sonata FLEXsoft	65.7	OPUS 2	8.7	6.2	**80**	55
BL 8	Concerto FLEX28	47.1	OPUS 2	3.4	3.4	**75**	65
BL 9	Concerto FLEX28	27.3	OPUS 2	3.6	2.3	**90**	35
BL 10	C40+ Standard	30.0	SONNET	14.4	14.6	**100**	100
BL 11	Synchrony FLEX28 Concerto FLEX28	63.2	OPUS 2/SONNET	1.0	3.6	60	**80**
BL 12	C40+ Standard	19.2	OPUS 2	15.3	17.4	**95**	85
BL 13	Concerto FLEX28	41.5	OPUS 2	3.9	3.9	**80**	70
BL 14	Concerto FLEX28	66.3	OPUS 2/SONNET	2.0	3.6	80	**95**

**Table 3:** Data of the bimodal EAS group. FMS: Freiburg monosyllable score. PTA: pure-tone average of frequencies 0.5/1/2/4 kHz. PTA_low_: pure-tone average of frequencies 125/250/500 Hz. Used hearing aids were from manufacturers Phonak (Stäfa, Switzerland, Oticon (Smørum, Denmark), and Siemens (Erlangen, Germany).

ID	Implant Type (Ear)	Age [yrs]	Sound Processor	EAS Listening Experience [yrs]	Hearing Aid Type	FMS Score EAS/Contra [%]	PTA EAS/Contra [dB HL]	PTA_low_ EAS [dB HL]
EAS_BM1	Sonata FLEX24 (left)	55.8	SONNET EAS	5.3	Phonak Naida S IX UP	70/70	117.5/81.3	73.3
EAS_BM2	Sonata FLEX24 (right)	53.5	SONNET EAS	5.8	Phonak Piconet 2 P2 AZ	85/20	102.5/101.3	43.3
EAS_BM3	Sonata FLEX20 (right)	73.8	SONNET EAS	5.9	SONNET (CI)	65/55	86.3/94	61.7
EAS_BM4	Synchrony FLEX24 (left)	70.8	SONNET EAS	2.0	Oticon Chili SP9	70/30	82.5/76	71.7
EAS_BM5	Synchrony FLEX24 (right)	59.7	SONNET EAS	4.1	SONNET (CI)	85/80	107.5/120	53.3
EAS_BM6	Concerto FLEX24 (right)	63.4	SONNET EAS	4.4	Siemens Nitro	95/35	98.8/84	38.3
EAS_BM7	Sonata FLEX24 (right)	52.7	SONNET EAS	6.8	-	85/25	101.3/110	31.7
EAS_BM8	Concerto FLEX24 (right)	51.3	OPUS 2 EAS	4.1	Siemens Motion P	50/30	98.8/98	51.7

**Table 4:** Data of the bilateral EAS group. FMS: Freiburg monosyllable score; ipsilateral ear for noise presentation in S0N60 test condition is indicated bold. PTA: pure-tone average of frequencies 0.5/1/2/4 kHz. PTA_low_: pure-tone average of frequencies 125/250/500 Hz.

ID	Implant Type (Left/Right)	Age [yrs]	Sound Processor (Left/Right)	EAS Listening Experience Left [yrs]	EAS Listening Experience Right [yrs]	FMS Score Left/Right [%]	PTA L/R [dB HL]	PTA_low_ L/R [dB HL]
EAS_BL1	Synchrony FLEX24	72.9	SONNET EAS	1.1	1.9	**100**/80	106.3/93.8	66.7/46.7
EAS_BL2	Sonata FLEX20/FLEX24	73.9	OPUS 2 EAS	6.2	7.6	70/**80**	106.3/100	85/73.3
EAS_BL3	Sonata FLEX24 Synchrony FLEX24	60.5	DUET 2 SONNET EAS	5.1	1.1	**85**/70	120/108.8	81.7/48.3
EAS_BL4	Concerto FLEX24	47.8	SONNET EAS	2.6	4.2	**100**/70	77.5/106.3	16.7/53.3
EAS_BL5	Concerto FLEX24 Sonata FLEX24	68.1	DUET 2 SONNET EAS	3.6	5.0	**75**/65	112.5/111.3	68.3/56.6
EAS_BL6	Synchrony FLEX24	45.6	SONNET EAS	0.2	0.3	**90**/80	106.3/88.8	23.3/21.7

## Data Availability

The raw data supporting the conclusions of this article will be made available by the authors on request.
